# Mechanism of Bacterial Signal Transduction Revealed by Molecular Dynamics of Tsr Dimers and Trimers of Dimers in Lipid Vesicles

**DOI:** 10.1371/journal.pcbi.1002685

**Published:** 2012-09-20

**Authors:** Benjamin A. Hall, Judith P. Armitage, Mark S. P. Sansom

**Affiliations:** Oxford Centre for Integrative Systems Biology, Department of Biochemistry, University of Oxford, Oxford, United Kingdom; Wellcome Trust Sanger Institute, United Kingdom

## Abstract

Bacterial chemoreceptors provide an important model for understanding signalling processes. In the serine receptor Tsr from *E. coli*, a binding event in the periplasmic domain of the receptor dimer causes a shift in a single transmembrane helix of roughly 0.15 nm towards the cytoplasm. This small change is propagated through the ∼22 nm length of the receptor, causing downstream inhibition of the kinase CheA. This requires interactions within a trimer of receptor dimers. Additionally, the signal is amplified across a 53,000 nm^2^ array of chemoreceptor proteins, including ∼5,200 receptor trimers-of-dimers, at the cell pole. Despite a wealth of experimental data on the system, including high resolution structures of individual domains and extensive mutagenesis data, it remains uncertain how information is communicated across the receptor from the binding event to the downstream effectors. We present a molecular model of the entire Tsr dimer, and examine its behaviour using coarse-grained molecular dynamics and elastic network modelling. We observe a large bending in dimer models between the linker domain HAMP and coiled-coil domains, which is supported by experimental data. Models of the trimer of dimers, built from the dimer models, are more constrained and likely represent the signalling state. Simulations of the models in a 70 nm diameter vesicle with a biologically realistic lipid mixture reveal specific lipid interactions and oligomerisation of the trimer of dimers. The results indicate a mechanism whereby small motions of a single helix can be amplified through HAMP domain packing, to initiate large changes in the whole receptor structure.

## Author Summary

To understand cell signalling events requires a physical model of the structure and behaviour of the signalling proteins involved. The methyl-accepting chemoreceptor proteins direct bacterial movement towards food sources and away from toxins. Based on experimental data we have built structural models of the serine chemoreceptor (Tsr) as a dimer, which is incapable of activating the downstream kinase CheA, and as a trimer of dimers, which can activate CheA. We have performed molecular dynamics simulation to reveal the behaviour of these two forms in a planar lipid bilayer and in a 70 nm diameter lipid vesicle with a mixture of lipids mimicking the *E. coli* inner membrane. We show that in isolation the dimers undergo a bending movement around the central HAMP domain, whereas the trimer-of-dimers model does not. Comparison with published experimental data suggests that these bending motions are real, and that they occur in the trimer of dimers only in response to ligand binding. Drawing together these observations with studies showing that the signalling event involves small piston motions in the transmembrane helices suggests that the bending motion is frustrated in the unliganded trimer of dimers, and that ligand binding induces bending by repacking the HAMP interface.

## Introduction

**Figure 1:**
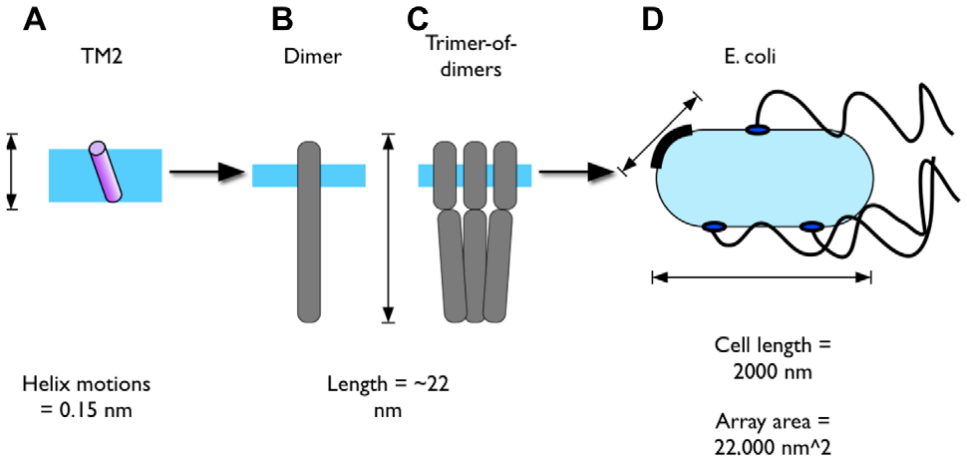
Schematic diagram of Tsr and chemosensing in *E. coli*. Scales in bacterial chemotaxis: **A** motions of a single helix in Tsr over ∼0.15 nm cause changes in the 22 nm scale structures of the receptor dimer **B** and trimer-of-dimers **C.** This in turn induces conformational changes in a hexagonal array **D** at the pole of the cell (with a surface area of 22000 nm^2^) which downstream (via diffusion of effectors along the 2000 nm cell length) results in a change the direction of rotation of the flagellar motors and the movement of the cell.

Transmembrane (TM) signalling plays a key role in cell biology. Signals can be conveyed across the membrane by subtle motions of single TM α-helices or TM helix dimers, and may result in substantial changes in protein structure and activity, and in turn lead to changes in cellular function and behaviour. One example where such signals are transmitted across a wide range of physical scales is the process of bacterial chemotaxis in *E. coli* (Figure 1, for a recent review see [1]). *E. coli* control their movement through motions of the flagellar motor; counter-clockwise (CCW) rotation of the motor causes the flagellar filaments to coalesce and the cell to move in a single direction (or “run”), whereas clockwise (CW) rotation causes the flagellum bundle to dissociate and the cell to reorient [2], [3], [4]. By varying the rate of switching between CW and CCW rotation of the motor, a cell can move away from toxins and towards nutrients, as well as responding to different aspects of its internal state.

The response to serine is mediated by the methyl-accepting chemoreceptor protein (MCP) Tsr. Tsr is one of four *E.coli* transmembrane chemoreceptors. These form stable homodimers which come together as trimers of dimers in a large receptor array at the cell poles. Serine binding to the periplasmic domain in Tsr causes small (∼0.15 nm) piston-like motions in the position of a single helix in the cytoplasmic membrane [5], [6]. This causes structural changes in the trimer-of-dimers structure, within a hexagonal receptor array [7]. Binding events of a single receptor in this array are amplified through a “conformational spread” across the 20,000 nm^2^ receptor array [8]. Through subsequent changes in the activity of the associated histidine kinase CheA induced by the changes in the receptor array activity (and the phosphorylation status of downstream effectors) the rate of flagellar motor switching and cell swimming behaviour is regulated.

The small conformational change induced by binding must be transmitted to a highly sensitive downstream signalling domain and amplified to create a conformational signal which can be passed through the highly stable receptor complex. In particular, the role of a highly conserved HAMP domain in the receptor is the topic of intensive study. HAMP is a linker domain present in histidine kinases, adenyl cyclases, methyl-accepting proteins and phosphatases which is found in bacterial sensor and chemotaxis proteins and in eukaryotic histidine kinases. High resolution structures have highlighted the importance of packing and orientation in isolated HAMP domains and poly-HAMP domains [9], and a range of genetic data has identified “on” and “off” HAMP mutations [10]. Furthermore the HAMP domains of different receptors can be experimentally swapped to produce functional receptors [9]. These results indicate that a mechanism of signalling through HAMP domains needs to be compatible with both systems which respond to piston motions (such as Tsr) and rotation signalling motions (such as NpHtrII [11]). A wide range of different, non-mutually exclusive models have been proposed to understand this general mechanism, including changes in HAMP stability [12], clockwork-like rotation of HAMP helices [9], and subtle changes in the helix crossing angle. Alongside this there are cryoelectron tomography data which indicate that these relatively subtle rearrangements lead to much larger changes in the trimer-of-dimers structure [7], and possibly also influence the array structure [13]. Extensive studies of isolated systems in nanodisc preparations, with different copy numbers, reveal that the trimer-of-receptor dimers is the fundamental kinase activating unit and remains responsive to ligand binding in isolation from the chemoreceptor array [14]. Individual dimers cannot be isolated in the bacterial membrane.

Here we describe the structure and predicted dynamic properties of full-length homology models of Tsr dimers (representing the non-kinase activating form) and trimers-of-dimers (representing the kinase activating form) using coarse-grained molecular dynamics (CG-MD) simulations. We explore the behaviour of these large assemblies over microsecond time periods, both in a model lipid membrane bilayer (DPPC), and also in a ∼70 diameter nm vesicle containing a mixture of cardiolipin, phosphatidyl-glycerol and phosphatidyl-choline and thus mimicking the *E. coli* inner membrane. CG-MD simulations have been used successfully to model a wide range of different phenomena relating to membrane proteins, including: transmembrane peptide anchoring, lipid-protein and lipid-DNA interactions [15], [16], [17]; protein-protein interactions [18]; [19]; and membrane protein conformational changes [20]. Thus, CG-MD compares favourably with both experimental data and with other theoretical approaches [21], [22], [23], [24], [25], and also allows for conversion to well configured starting models for atomistic MD simulations in a multiscale approach [26]. The reduction in detail in CG-MD approaches compared to atomistic representations increases the effective speed of simulations by 2–3 orders of magnitude, enabling larger simulations to be performed with significantly improved sampling [27], [28]. From these simulations we propose a mechanism of signalling through the HAMP domain by destabilisation of the helix interfaces through a piston motion. Furthermore, our results suggest that cardiolipin preferentially binds to the chemoreceptor dimers in the inner membrane through interactions with basic amino acid residues.

## Results

### Model of the Tsr dimer

**Figure 2:**
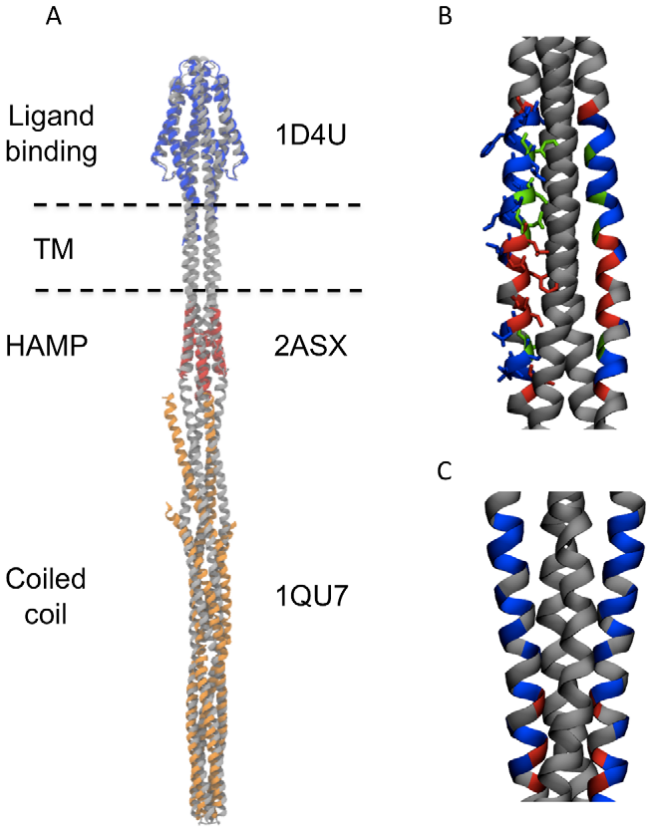
Initial model of Tsr dimer. **A** Initial symmetric dimer in grey, overlaid onto the aligned crystal structures (ligand binding domain in blue, HAMP domain in red, and coiled coil region in orange). AS2 helices are positioned on the left and right of the HAMP domain. (B) and (C) Disulphide mapping data, plotted as colours on the surface of the TM domain for **B** TM1 and **C** TM2. Red residues crosslink strongly, green residues crosslink to an intermediate extent, and blue residues do not crosslink. In both diagrams it can be seen that higher crosslinking efficiency residues face their dimeric partner, largely in agreement with experimental data (TM2 appears to match experimental data better than TM1).

Models of the complete length, symmetric dimer of Tsr were built from the crystal structures of component domains where available (2D4U, 1QU7), and for HAMP from the NMR structure (2ASX) of the homologous HAMP domain from *Archeoglobus fulgidus* [9] (Figure 2A).

We generated models of the dimers as described below. Briefly, models of pairs of domains connected by helices were generated and converted to their coarse grain representations. These were then simulated for 200 ns to allow the structure to equilibrate. The equilibrated structures were used as inputs alongside the high resolution structures to build the complete symmetric model of the dimer. The resulting structure was validated against a variety of experimental data, including the available high resolution structures from the PDB [9] (overlaid on the model in Figure 2A), disulphide mapping data (Figure 2B) and mutational data on residues known to interact specifically with the membrane, in the dimer of the related chemoreceptor Trg [29]. These show good agreement with the observed orientation of the transmembrane helices, where regions of higher linkage efficiency can be seen to interact (Figure 2B). Furthermore, extensive mutagenesis of conserved residues in the interfacial region of closely related proteins [30], [5], [6] indicate that conserved tryptophan residues interact with the membrane. This is consistent with the outward facing orientations of aromatic residues in our model.

### Dynamics of isolated Tsr dimers in model membranes

**Figure 3:**
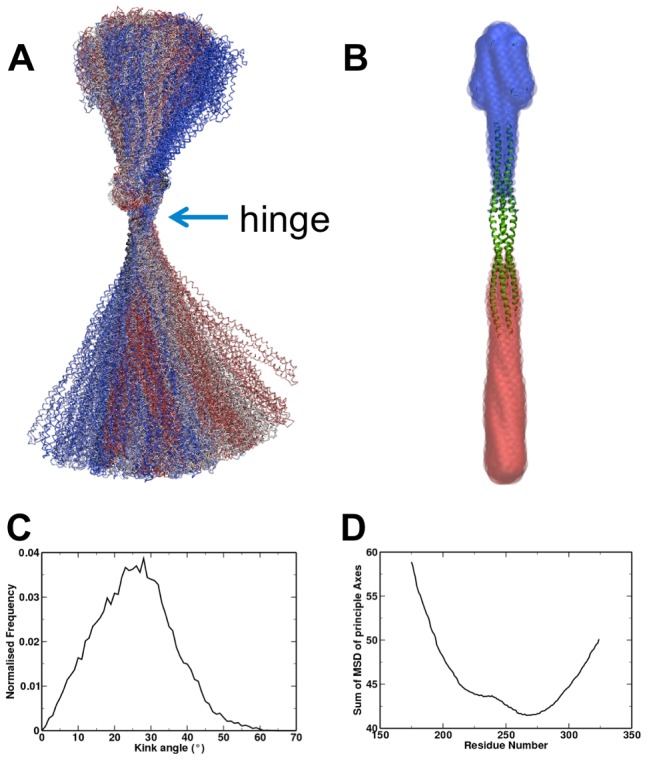
Dynamics of Tsr dimer. **A** The ensemble of structures from a single simulation, fitted on the hinge region (marked). Individual structures are coloured by timestep; there is no clear pattern in kinking over time. To find the hinge point of this bending motion we calculated the mean-square-deviation of CA positions from two lines of best fit of different bisections of the protein around the HAMP/coiled-coil domain interface. **B** First mode of a GNM analysis of the dimer model, indicating a bending motion around the same hinge observed in MD. The dominant eigenvector (consisting of a scalar value for each residue) is plotted as transparent sphere overlaid on the cartoon representation of the structure, with red/blue indicating positive/negative scalar values. The hinge region is identified from the area with small eigenvector values, following [58] **C** Histogram of bending angles from a single Tsr simulation. The modal angle is roughly 30°. **D** Bending analysis of the helix. 2 lines of best fit are generated from backbone particles above or below the identified residue, and the mean square deviation of fits of these 2 axes is calculated and plotted. The minimum represents the location of the bending hinge (i.e. the point where the backbone positions best fit a line).

Four simulations each of 5 microsecond duration were performed for the isolated Tsr dimer structure in a phospholipid (DPPC) membrane (see Table 1). In each simulation the structures were observed to retain their fold but undergo extreme bending motions around the HAMP/coiled-coil domain interface (Figure 3A), whilst retaining the fold of the constituent domains. The hinge residue was G268 (Figure 3C) and the structure was seen to bend by up to 60° over the course of the simulations, with a modal bending angle of 30° (Figure 3C). Throughout these motions, the folds of the individual domains are visibly maintained, showing modal backbone RMSDs of ∼0.4 nm for the coiled coil domain, ∼0.44 nm for the HAMP domain, and 0.65 nm for the ligand binding domain (noting that motions along the dimer interface increase the apparent RMSD). The HAMP domain retains its hydrophobic interface, but helix AS2 (the second helix of each monomer in the HAMP domain) leading to the coiled-coil domain can be seen to slide away from the membrane. This degree of bending motion was unexpected, so to confirm the predicted structural dynamics we also performed GNM and ANM analyses on the model structure [31], [32] (Figure 3D). These confirmed the presence of a hinge point around G268, showing bending motions around this region in the dominant modes of motion.

**Table 1:** Summary of simulations.

System	Temperature (K)	Duration
Dimer in DPPC bilayer	323	4×5 µs
Trimer of dimers in DPPC bilayer	323	0.5 µs*+1.0 µs
Vesicle**	323	0.5 µs
Vesicle**	295	0.5 µs
Vesicle** with 30 dimers	295	1.5 µs
Vesicle** with 10 trimers of dimers	295	1.5 µs

* ENM restraints were imposed between crystal contacts residues for the first 0.5 µs. See main text for details.

** The lipids in the vesicle were: DPPE∶DPPG∶CL = 7∶2∶1; see main text for details.

We re-examined several different experimental studies containing structural data on the behaviour of isolated chemoreceptor dimers to determine whether this bending is realistic. In Tar R259 (which corresponds to residue 261 in Tsr, neighbouring the kink region) has been highlighted as a region which is sensitive to proteolysis and therefore proposed as a region of high flexibility [33], [10]. In particular, this flexibility was highlighted as a region of possible unfolding, based on the inability of proteases to hydrolyse intact helices, and a hinge region between the discontinuous 4 helix bundles was proposed [10]. Nanodisc preparations with single chemoreceptor dimers visualised using electron microscopy show distinct kinking around the HAMP domain [14]. Finally, cryoelectron tomography data on the complete, ligand bound (kinase activating) trimer-of-dimers [7] would appear to require bending around the HAMP domain to connect the coiled-coil region to the transmembrane domain, although this is not seen in all cryoelectron tomography studies, provoking some discussion [34]. Taken together, these separate experimental approaches appear to support the bending of Tsr around a hinge point between the HAMP domain and the coiled-coil region. Previous crystal structures have also identified a glycine hinge at a different location in the coiled coil [35], though we have not observed any clear bending outside of the HAMP hinge.

Despite the significant extent of the bending observed the fold of the individual proteins is maintained. At the hinge point, however, the bending causes structural repacking in the HAMP domain. Specifically, the bending largely maintains the hydrophobic packing interface, but the AS2 helices (leading to the coiled-coil domain) undergo rotations and sliding throughout the simulation. Previous disulphide mapping studies on the related HAMP domain from Tar indicated that introduction of disulphide bonds into HAMP could lock the receptor into a kinase on/off state [10]. In Tar, the residues A231C-H256C′ (corresponding to A233/H238′ in Tsr, with a prime notation indicating the opposing chain) induce a lock-on effect upon introduction of a disulphide bond. In contrast to this, the mutations I224C-T253C′ (corresponding to L226/S255′ in Tsr) induce a lock-off state when a disulphide bond is introduced. In these simulations, the lock-off residues are adjacent throughout the simulation, whereas the lock-on residues move away from their original, nearby positions in the model to face away from one another. Taken together, these experimental data suggest that our prediction of a highly bent structure is realistic, and closely reflects a kinase-inactive state for the protein. This is in agreement with activity assays of isolated Tsr dimers in nanodiscs [36].

### Dynamics of a model of trimer-of-dimers in a model membrane

**Figure 4:**
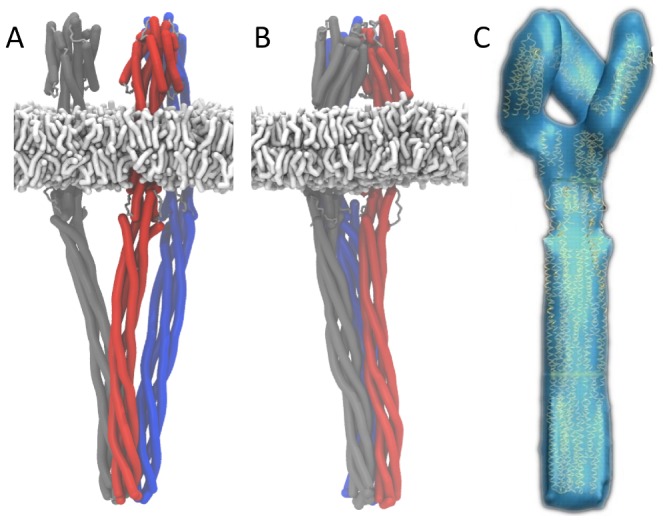
Models of the Tsr trimer of dimers. **A** Initial trimer-of-dimers model based on a structure exhibiting the modal bending angle derived from simulations of the individual dimers, with the constituent dimers packed together into the trimer using crystal contacts in the coiled coil domain (1QU7). **B** “Closed” trimer model generated after 0.5 µs MD with restraints on crystal contacts. **C** Cryoelectron tomography image of the trimer taken from Khursigara et al [7].

We next built a complete trimer-of-dimers model using snapshots taken from simulation of Tsr dimers (with a bending angle of ∼30°). Crystal contacts were used to model the trimer of dimers interface (Figure 4A), as used by Shimizu et al. [37]. To retain these contacts over the simulation, elastic network model-like restraints were imposed between crystal contact residues for the first 0.5 µs of the 1.5 µs simulation (Table 1). Over this initial time period the structure rapidly contracted to form a single rod like structure without large gaps between the coiled coil domains (Figure 4B). This structure was then simulated for a further 1 µs, without the crystal contact restraints during which period the structure remained relatively stable, and did not show the extreme bending observed for the isolated dimers, due to the stabilising effect of trimerization. To the best of our knowledge, such stabilisation of Tsr upon trimerisation has not been suggested previously, and may help to explain the difference in kinase activation ability between the trimeric and monomeric forms. The resulting structure closely resembles the observed low resolution cryoelectron tomography electron density of the apo form (Figure 4C, used with permission from [7]), further validating the model.

The dynamics of the trimer-of-dimers assembly is distinct from the behaviour of the dimer in that trimer-of-dimers structure remains more stable and does not exhibit the pronounced bending that was observed for the dimer (and so also does not show the associated HAMP rearrangement). Experimental preparations of Tsr in nanodiscs have shown that the complete trimer of dimers is required for kinase activation [38], [39], and cryoelectron tomographic data indicates that a distinct conformational change is undergone by HAMP upon ligand binding [7]. Together, we propose that this more stable structure represents the kinase active form. One unexpected result is the tight packing along the adaption region, which excluded most of the CG water particles. Of course, such exclusion of water may in part reflect the approximations of the CG model. It is possible that in an atomistic model some water molecules might be able to form part of this interface. In our model the cooperative zipping of the structure stabilises the packing, and it may be that the charges of acidic residues in different methylation states destabilise the zipping interactions as part of the adaption process.

### Dynamics of Tsr dimers and trimers-of-dimers in a 70 nm lipid vesicle

**Figure 5:**
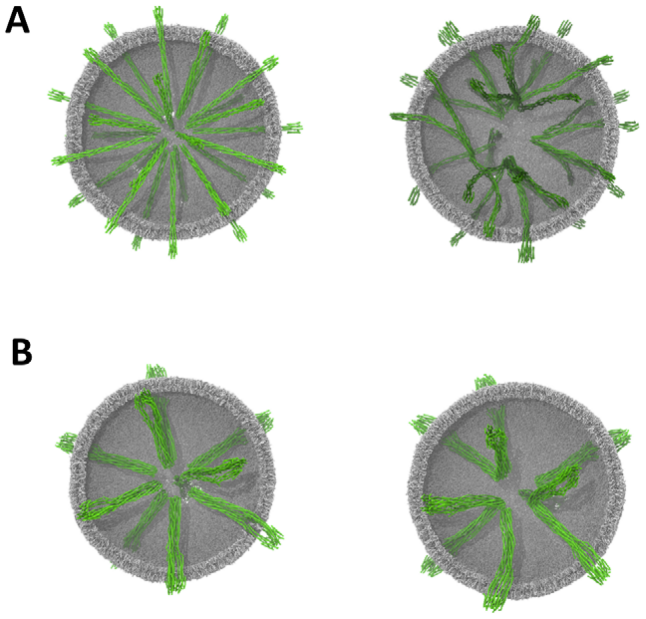
Simulations of multiple Tsr dimer models in a biologically realistic membrane in the form of ∼70 nm diameter lipid vesicles. Helices are rendered as green curved tubes, lipids are in grey. **A** Tsr dimers at t = 0 and 1.5 µs, and **B** Tsr trimer-of-dimers at t = 0 and 1 µs. The Tsr dimers can be observed to make a wide variety of interdimer contacts, whereas the trimers-of-dimers form only limited interactions with one another.

Simulations of multiple Tsr dimers and trimers-of dimers (the latter from the final frame of simulations of the restrained trimer-of-dimers) were performed over at least 1 microsecond, in a 70 nm lipid vesicle. The lipid composition of the vesicle was intended to mimic that of the native *E. coli* inner membrane lipid bilayer and consisted of a mixture of 10% cardiolipin, 18% DPPG and 72% DPPE. 30 dimers or 10 trimers-of-dimers were distributed evenly across the surface of the vesicle and the structures were free to interact without steering or restraints (Figure 5; Table 1).

**Figure 6:**
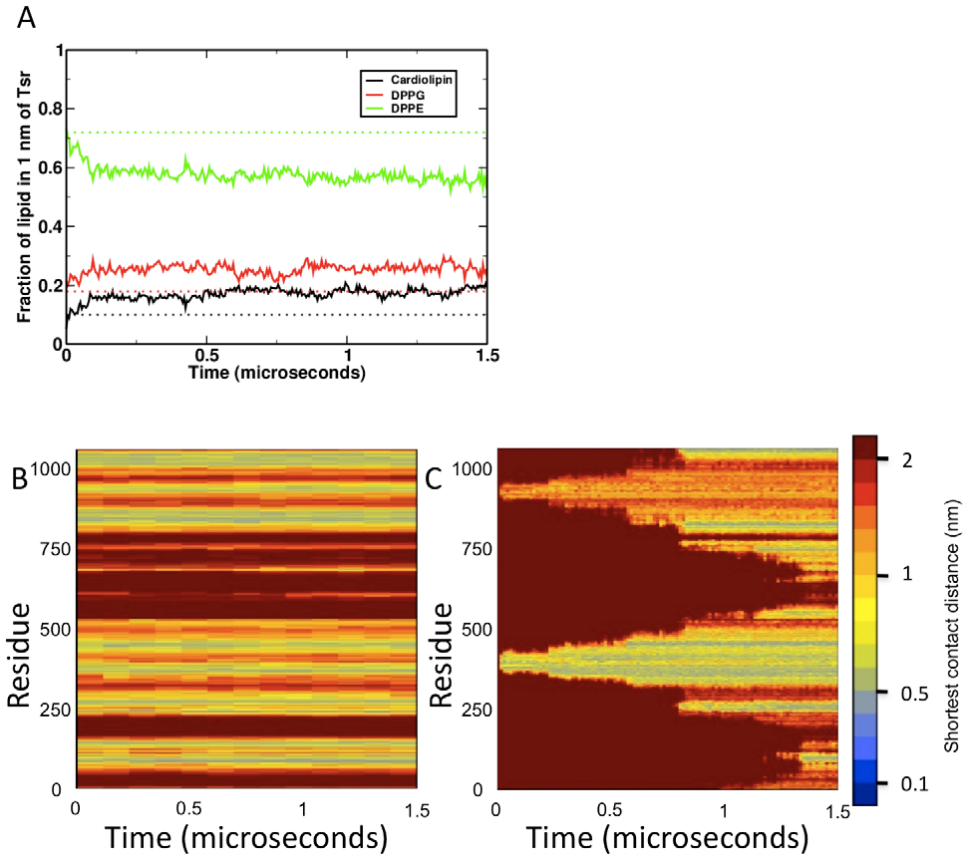
Interactions of proteins with their environment over time. (A) Specific enrichment of anionic lipids (cardiolipin and DPPG) within a 1 nm distance of the proteins over time, relative to DPPE. (B) and (C) Example interaction fingerprints from trimer-of-dimers and dimer vesicle simulations. These show the shortest distance between a given residue in one dimer and any residue in the binding partner over time (based on the approach in [19]). (B) shows dimer-dimer interactions within a trimer-of-dimers, and (C) shows dimer-dimer interactions which appear over the course of the simulation. Over time, the interaction surface in (C) changed to mimic the stable, unchanging binding surface of the trimer-of-dimers model (B), indicating that the structure naturally oligomerises along this interface.

Previously observed kinking behaviour of the Tsr dimers was replicated in the vesicle system, where the bending motions allowed individual dimers to form interactions with other dimers over the simulation. Over 1.5 microseconds some of these inter-dimer interactions (Figure 5A) can be seen to mimic the extensive contacts observed in the trimer-of-dimers (Figure 6). In contrast to the isolated dimers, the trimers-of-dimers form fewer and less extensive interactions over the course of 1 microsecond (Figures 5B), and show reduced bending around the HAMP domain than the dimeric Tsr. These non-specific interactions are partially supported by a range of different studies which show that overexpression of the chemoreceptors can lead to extensive intra-dimer interaction and the creation of unusual membrane structures [40]. Furthermore studies of the related system McpG demonstrated that in the absence of CheA/CheW loose association of the receptors were able to associate and specifically localise at the cell poles in *R. sphaeroides* [41].

The mixture of lipids has been shown to be important for function of the Tsr trimer of dimers in nanodisc preparations. Cardiolipin is known to locate to the pole of the cell [42], where the receptor array is located, and to a specific point of high local curvature in spheroblasts prepared from the *E. coli* inner membrane [43]. Certain lipid mixtures at room temperature have furthermore been shown to undergo phase separation in coarse grained simulations [44], [23]. The empty vesicle did not show any evidence of lateral phase separation at either 295 K or 323 K, with lipids being evenly distributed across the vesicle surface. In contrast to this, the presence of both Tsr dimers and trimers-of-dimers can be seen to cause a local enrichment of anionic lipids within a 1 nm distance of the protein (Figure 6). Cardiolipin specifically can be seen to double in concentration under these conditions, and to make close contacts between its phosphate headgroup and basic residues in the inner membrane leaflet. Additionally, the lack of phase separation in vesicles without proteins suggests that cardiolipin moves to the cell pole through specific interactions with proteins rather than through a preference for regions of high membrane curvature. It is also possible that the formation of a hexagonal array would supplement the clustering effect and may be able to induce phase separation at room temperature by creating an area of the membrane with a sufficiently high local cardiolipin location. It is noteworthy that the curvature in this vesicle is significantly higher than the curvature of the *E. coli* cell poles, and it is possible that this may alter the signalling behaviour by altering the packing of lipids against the protein surface.

## Discussion

In this study we have built and simulated the dynamics of complete models of the bacterial receptor Tsr using coarse grained molecular dynamics, in different membrane environments. We have found that Tsr dimers in isolation undergo large bending motions around the region connecting the HAMP domain to the coiled coil domain, which is supported by experimental data and unlikely to represent a kinase activating structure (i.e. not the signalling state). Models of the trimer of dimers state show the stabilising effects of the trimer assembly, and that the trimer does not undergo the same bending. This trimeric form resembles the observed cryoelectron data and likely represents the kinase activating form. The bending observed in the isolated dimer, in the context of the trimer-of-dimer can be expected to relate the observed cryoelectron density of the kinase active apo trimer-of-dimers to the ligand bound form, with observed “expanded” structure. EPR data on the structure and dynamics of HAMP domains in the related signal transducer NprHtrII showed that in the absence of light of the HAMP domain of the protein also adopted a “dynamic” state, where the packing of the domain was rearranged and mobility increased [45], which would support our observations both in terms of the structure and dynamics observed. These behaviours indicate that signalling through small piston motions in the helix TM2 could induce bending around the HAMP domain. Given the unusual “knobs-to-knobs” packing observed in the HAMP structure, we speculate that piston motions induce bending by altering the helix packing within the domain, inducing bending and trimer expansion, leading to a loss in kinase activation. Finally, in a mixed lipid vesicle, we observe dimers oligomerising at equilibrium to form extensive contacts observed in our model trimer-of-dimers and the specific clustering of anionic lipids (cardiolipin and PG) around the protein.

Overall, these motions suggest that the HAMP interface is sensitive to small motions and able to undergo changes in response to these motions to the complete protein. Signalling events in the periplasm have been proposed to cause a piston-like motion of TM2, based on a wide range of experimental and computational studies (for a recent review see [1]), and would be expected as such to disrupt the unusual “knobs-to-knobs” HAMP interface. We propose on the basis of these data and our models that in Tsr a piston-like motion causes a destabilisation of the HAMP interface and alters the bending properties of the entire receptor dimer. This is in agreement with the observed cryoelectron tomography data on the ligand bound receptor, which requires a bending around HAMP to connect the coiled-coil domain to the transmembrane domain [7]. Additionally, a similar model for signalling through the HAMP domain has been previously proposed based on disulphide mapping data [10], [12]. Such a mechanism would be compatible with the different mechanisms of signal propagation through the membrane (i.e. helix rotation or piston) as long as such motions lead to a destabilisation in of the HAMP packing. It is interesting to note that the bending region occurs both in a region with both the unusual helix packing of the HAMP domain, but also the only region of the protein where four helices are not continuous (due to the loops in the HAMP domain). Given the rod like structure of the dimer, it is intriguing to speculate that rod like structures, which can be expected to bend more or less about their centre, may be tuned in biology to bend in specific locations by the introduction of more malleable regions, either by frustration (eg the knobs to knobs packing) or by breaks in secondary structure (eg the HAMP loops).

A key target for future studies would be to attempt to explicitly induce expansion of the trimer by modelling the signal transduction event. This would require us to extend the simulations to explicitly model proposed conformational changes that occur on ligand binding, in particular the proposed piston shift of TM2 [1], [21]. Such approaches would require either very large scale atomistic simulations [46] or modifications to the CG model to allow more subtle treatments of conformational changes [47]. In this way it should be possible to provide detailed insights into an experimentally hard to access system, and to confirm the degree of motion undergone by different components in the macromolecule.

The role for lipids in the bacterial membrane in chemotactic signalling and other systems is a growing area of study. Cardiolipin is known to localise at the cell poles and the poles of spheroblasts derived from the *E. coli* inner membrane [43]. Experimental and simulation data on the interaction of proteins with lipids in mixed membranes suggest that proteins can both influence domain formation and respond to phase partitioning by moving into preferred regions [23]. We have found that phase formation does not occur in our model vesicle system in the absence of protein, but in the presence of the bacterial chemoreceptors cardiolipin (and to a lesser extent phosphatidyl-glycerol lipids) clusters around the protein, interacting specifically with charged residues in the inner membrane. The higher curvature of the vesicle compared to the bacterial membrane would be expected to induced phase formation on its own if this drove cardiolipin pooling at the cell pole. This would suggest that in a larger system, where the chemoreceptor proteins are tightly packed in an orderly array, the proteins themselves may be able to induce the observed cardiolipin partitioning. This may be enhanced if phase formation for cardiolipin could be induced by reaching a critical threshold local concentration. The unnaturally high curvature itself may play a role in inducing protein interactions; whilst bending in the dimer could still be expected to lead to protein-protein interactions, the reduced bending observed in the trimer-of-dimers may not allow for this.

This study demonstrates the importance of modelling large, macromolecular systems in interpreting experimental evidence and in making predictions on the role and interactions of different structural components. The signalling process passes through small changes in the periplasmic binding and transmembrane domain to large changes across a massive macromolecular array. Simulations of models of the complete dimer and trimer-of-dimers have allowed us to propose a mechanism of signalling through the chemoreceptor and to validate them against a wide range of distinct sources of experimental data. Specifically we observe motions in the isolated dimers which are not observed in the trimers of dimers, due to constraints imposed by dimer-dimer contacts, which reflect the difference between signalling and non-signalling forms of the receptor. Simulations in a mixed lipid vesicle have enabled us to extend this to examining the specific lipid contacts and the oligomerisation of individual dimers at equilibrium. As such, this approach may be used in future to develop and validate detailed models of the larger receptor array contacts with other key components such as CheA, CheB and CheR.

## Methods

### Models of Tsr

An intitial dimeric model of Tsr was built from the individual structures of domains (or homologous domains) available (PDBID: 1QU7, 2D4U, and 2ASX) and equilibrated domain pair structures (receptor/transmembrane, HAMP/coiled-coil). To build an initial approximation of the inter-domain regions we built models based on pairs of domains connected by continuous helices (transmembrane domain/receptor domain, HAMP domain/coiled coil domain). These were modelled as helices as both PSIPRED and Jpred3 predictions predict continuous helicity in the missing regions [48], [49].

These structures were converted to coarse grain models using standard techniques as described below, and simulated for 200 ns at 323 k in cuboid boxes with explicit CG water and counter-ions to neutralise the system. The missing regions were explicitly modelled as helices during the coarse grain process, enforcing expected secondary structure. Over the course of the receptor/TM domain pair simulation, the TM helices could be observed to assemble from a loose, splayed conformation into a tight bundle, without lipids trapped between the helices.

The resulting trajectories from these simulations were visualised and representative structures from the last 100 ns identified through clustering using g_cluster (using the GROMOS algorithm). The middle structures of the dominant cluster identified in each simulation was converted back to atomistic models using the approach of Stansfeld et al [26], and used as new inputs for building the complete model of the dimer. All structures were aligned in space based on their carbon alpha atoms prior to their use by Modeller, exploiting overlaps between the different structures. The final models used for simulation were built from the high resolution structures and overlapping connecting regions taken from the domain pair simulations.

The individual high resolution structure of the domains were each symmetrical (or near symmetrical) in their observed forms. Furthermore, the regions modelled in the domain pair simulations packed together tightly (excluding water/lipids where 4 helices were able to pack together), and becoming near symmetrical. Thus, in building the model of the complete Tsr dimer, symmetry was imposed on the complete dimer, and helicity was imposed in the connecting regions. 25 models were generated with Modeller [50], and the model with the lowest objective function selected. This model was converted to CG and used for production simulations.

To generate a trimer of dimers model of Tsr, snapshots from CG-MD simulations of the Tsr dimer, and the crystal contacts observed in PDB:1QU7. The resulting structure was initially simulated for 500 ns with harmonic restraints in the trimerisation region between individual dimers.

### Coarse grained molecular dynamics

CG-MD simulations were performed using procedures described previously using the standard MARTINI forcefield [51], [28], [52]. Briefly, 4∶1 mapping of non-H atoms to CG particles was used. Inter-particle interactions were treated with Lennard Jones interactions between 4 classes of particles; polar (P), charged (Q), mixed polar/apolar (N) and hydrophobic apolar (C). These were then split into subtypes to reflect differing hydrogen bonding capabilities or polarity. In the MARTINI forcefield, N and Q classes were subdivided into 5 and 4 subtypes respectively to reflect hydrogen bonding capabilities. Additionally P and C particle types were subdivided to reflect varying degrees of polarity. Interactions in each case were based on a lookup table, with 9 levels in MARTINI. Electrostatics were treated Coulombically. Protein secondary structure was maintained through dihedral restraints (elastic network models were not used to restrain the structure for production simulations). Lennard Jones interactions were shifted to zero between 0.9 and 1.2 nm, and electrostatic interactions were shifted to zero between 0 and 1.2 nm.

Simulations were performed with Gromacs 4 (www.gromacs.org) [53]. Temperature was coupled using a Berendsen thermostat at 323 K or 295 K (tau_t = 1 ps), and pressure was coupled semi-isotropically (across XY/Z) for bilayer simulations and anisotropically for vesicle simulations, at 1 bar (compressibility = 3×10-5 bar-1, tau_p = 10 ps) using the Berendsen barostat. All simulations performed in this study are listed in supplementary table 1. Helical secondary structure was maintained using dihedral restraints (60°, 400 KJ/mol), and all other (coil) regions were treated without dihedral restraints, following the MARTINI approach (as there are no β-sheet regions). No further restraints (ie elastic network restraints) were used in analysed simulations. In the generation of the trimer of dimers model, crystal contacts were restrained by harmonic restraints (1000 KJ/mol). Basic residues near the membrane were treated as charged, based on experimental evidence on the behaviour of model peptides containing lysine and arginine residues [54], [55], [56], [17].

### Elastic Network Modelling

Gaussian and anisotropic network modelling was performed as described in [31]. Briefly, all carbon alpha atoms within a given distance cut off are considered as connected by springs of equal strength. A cut off of 0.7 nm was used for the GNM and 1.3 nm for the ANM.

### Vesicle preparation

A ∼70 nm vesicle was generated by self assembly from a loosely packed shell generated with using packmol [57] at 323 K in a dodecahedral box under anisotropic pressure coupling, over 100 ns. In total 35,000 lipid molecules were used, including 3500 Di-PO-cardiolipin molecules, 7000 DPPG molecules, and 24,500 DPPE molecules. [57] The vesicle was solvated and counter ions added, and allowed to form by free self assembly, and equilibrated for 100 ns.
